# Characterizing the Xenoma of *Vairimorpha necatrix* Provides Insights Into the Most Efficient Mode of Microsporidian Proliferation

**DOI:** 10.3389/fcimb.2021.699239

**Published:** 2021-06-16

**Authors:** Tian Li, Zhuoya Fang, Qiang He, Chunxia Wang, Xianzhi Meng, Bin Yu, Zeyang Zhou

**Affiliations:** ^1^ State Key Laboratory of Silkworm Genome Biology, Southwest University, Chongqing, China; ^2^ Chongqing Key Laboratory of Microsporidia Infection and Control, Southwest University, Chongqing, China; ^3^ College of Life Science, Chongqing Normal University, Chongqing, China

**Keywords:** microsporidia, *Vairimorpha necatrix*, xenoma, subcellular structure, proliferation

## Abstract

Microsporidia are a group of obligated intracellular parasites that can infect nearly all vertebrates and invertebrates, including humans and economic animals. Microsporidian *Vairimorpha necatrix* is a natural pathogen of multiple insects and can massively proliferate by making tumor-like xenoma in host tissue. However, little is known about the subcellular structures of this xenoma and the proliferation features of the pathogens inside. Here, we characterized the *V. necatrix* xenoma produced in muscle cells of silkworm midgut. In result, the whitish xenoma was initially observed on the 12^th^ day post infection on the outer surface of the midgut and later became larger and numerous. The observation by scanning electronic microscopy showed that the xenoma is mostly elliptical and spindle with dense pathogen-containing protrusions and spores on the surface, which were likely shedding off the xenoma through exocytosis and could be an infection source of other tissues. Demonstrated with transmission electron microscopy and fluorescent staining, the xenoma was enveloped by a monolayer membrane, and full of vesicle structures, mitochondria, and endoplasmic reticulum around parasites in development, suggesting that high level of energy and nutrients were produced to support the massive proliferation of the parasites. Multiple hypertrophic nuclei were found in one single xenoma, indicating that the cyst was probably formed by fusion of multiple muscle cells. Observed by fluorescence *in situ* hybridization, pathogens in the xenoma were in merongony, sporogony, and octosporogony, and mature stages. And mature spores were pushed to the center while vegetative pathogens were in the surface layer of the xenoma. The *V. necatrix* meront usually contained two to three nuclei, and sporont contained two nuclei and was wrapped by a thick membrane with high electron density. The *V. necatrix* sporogony produces two types of spores, the ordinary dikaryotic spore and unicellular octospores, the latter of which were smaller in size and packed in a sporophorous vesicle. In summary, *V. necatrix* xenoma is a specialized cyst likely formed by fusion of multiple muscle cells and provides high concentration of energy and nutrients with increased number of mitochondria and endoplasmic reticulum for the massive proliferation of pathogens inside.

## Introduction

Microsporidia are obligate intracellular parasites and composed of at least 200 genera and 1,400 species (Fayer and Santin-Duran, 2014). Microsporidia can infect nearly all animals, including humans and economically important animals like silkworm, bee, shrimp, crab, and fish (Franzen and Müller, 1999; Joseph et al., 2006). The life cycle of microsporidia can be generally divided into three phases, the initially infective phase, proliferative phase, and sporogonic phase (Cali et al., 2005; Joseph et al., 2005). The first phase is the only stage that exists outside of the host cells, while the latter two phases must be inside host cells (Han and Weiss, 2017).

Different microsporidia species lead to varieties of symptoms and proliferate in divergent patterns. Most microsporidian infections usually cause no obvious tissue lesions, especially for those that can be vertically transmitted, while some species like *Vairimorpha necatrix*, *Glugea arabica*, *Vavraia lutzomyiae*, and *Potaspora morhaphis* can produce xenoma, which is a cyst full of pathogens and presents in many infected insects and aquatic animals (Lom and Dykova, 2005; Matos et al., 2006; Casal et al., 2008; Meng et al., 2018). In infected tissues, microsporidia and host cells interact and form a well-organize xenoparasitic complex, which was finally named “xenoma” by Weissenberg in 1949 (Lom and Dykova, 2005). The *G. arabica* could infect the intestinal wall of the marine teleost *Epinephelus polyphekadion* and produce spherical blackish xenomas (Azevedo et al., 2016). It was found that the xenoma of *Abelspora portucalensis* was scattered in the hepatopancreas of the common foreshore crab and more often observed at the edges of this organ. Most xenomas are formed by fusion of host cells and consist of hypertrophic cells (Azevedo, 1987). A real xenoma was pointed out to be a swollen host cell and surrounded by collagen fibers produced by the host (Cali and Takvorian, 1999). In a xenoma, host nucleus undergoes amitosis to form many small nuclei, and host organelles increase significantly, including mitochondria and endoplasmic reticulum (ER) (Cali et al., 2012).

Microsporidian *V. necatrix* was originally isolated from *Pseudaletia unipuncta* (Kramer, 1965; Pilley, 1976), and is primarily a pathogen of phytophagous Lepidoptera, including at least 36 insects (Maddox et al., 1981). *V. necatrix* is considered to be a potential insecticides for its wide host rang and high virulence (Down et al., 2004). We obtained a *V. necatrix* isolate, named *V. necatrix* BM, from the naturally infected silkworm (Liu et al., 2012; Luo et al., 2014). The infected silkworm presented typical symptoms similar to pébrine disease that caused by *Nosema bombycis*. The silkworm midgut, fat body, and testis were seriously infected, and silk gland and malpighian tubes were slightly infected, while the ovary could not be infected, suggesting that *V. necatrix* cannot be transovarially transmitted in silkworm (Meng et al., 2018). In particular, some xenomas containing massive pathogens were produced on the midgut, manifesting the importance of xenoma for the proliferation of *V. necatrix*. We have characterized the morphology of *V. necatrix* xenoma in our previous work (Meng et al., 2018). However, little is known about its subcellular features, as well as the development of parasites inside. Here, we dissected the *V. necatrix* xenoma by taking advantage of the microsporidia-silkworm system. Silkworm *Bombyx mori* is an ideal model to study lepidopteran insects and their pathogens.

## Materials and Methods

### Preparation of *V. necatrix* BM Spores

*V. necatrix* BM was isolated from the infected silkworm in Shandong Province, China. The fresh *V. necatrix* BM spores were purified from infected silkworms as described earlier (Liu et al., 2012). After removing the intestinal and puparium from the infected silkworm pupa, tissues were ground in sterilized distilled water using a mortar. The lapping liquid was filtered using cotton to remove tissue fragments and collect the effluent liquid, which was centrifuged at 5,000 rpm for 5 min at 4°C. After removing supernatant, the precipitate was washed with sterilized distilled water for three times, and resuspended with sterilized distilled water. The resuspended spores were counted with hemocytometer and stored at −80°C.

### Silkworm Infection

The eggs of silkworm *B. mori* Dazao were obtained from the State Key Laboratory of Silkworm Genome Biology, Southwest University, China. Silkworms in third instar were orally inoculated with 10^5^ V*. necatrix* BM spores per larva, and then reared to pupa stage. The infection was observed by dissecting analysis of the larvae in late fifth instar. Silkworm tissues were fixed with 1 ml of 2.5% glutaraldehyde and 4% poly-formaldehyde. Pathogen load in the tissues was then counted under ordinary optical microscope.

### Scanning Electron Microscopy (SEM) Assay

The SEM assay was performed referring to (Schottelius et al., 2000). Xenomas were fixed with 2.5% glutaraldehyde and 1% osmium tetroxide, and dehydrated with gradient ethanol (30, 40, 50, 60, 70, 80, 90, and 96%) for 10 min each, and 100% ethanol for two times for 15 min each. Then, the samples were dehydrated with gradient tert-butyl alcohol (50, 75, and 100%), and tert-butyl alcohol: acetonitrile (2:1 and 1:1), followed by absolute acetonitrile for 10 min each. Finally, the dried samples were coated with gold and observed using SEM S-3000N.

### Paraffin Sections and Confocal Observations

Silkworm midgut and xenoma were fixed in 4% paraformaldehyde and 0.1% glutaraldehyde and embedded in paraffin wax. The samples were then cut into 5 µm slices and placed on the slides. After deparaffinization and hydration, sections of the slides were stained with hematoxylin and eosin (HE) (Fischer et al., 2008). The other sections of the slides were incubated with DAPI and Fluorescent Brightener 28 (Sigma) at 37°C for 15 min. The slides were then washed for three times with 0.01M PBS buffer (pH 7.2) and suspended with Fluoromount™ Aqueous Mounting Medium (Sigma) and mounted with a cover glass. Finally, the slides were observed and photographed using an OLYMPUS Biological Confocal Laser Scanning Microscope FV1200.

### Staining the Nucleus, Mitochondria, and ER of Xenoma

Xenomas were fixed in 4% paraformaldehyde, decolorized with 6% H_2_O_2_ in ethanol for 2 h, and washed four times (10 min each) with 0.01 M PBS buffer (pH 7.2). The fixed samples were then incubated with DAPI, Mito-Tracker Red, and ER-Tracker Red at 37°C for 30 min to stain the nucleus, mitochondria, and ER, respectively. After washing four times (5 min each) with 0.01 M PBS buffer (pH 7.2), the slides were suspended using Fluoromount™ Aqueous Mounting Medium (Sigma) and mounted with a cover glass. The slides were finally observed and photographed using an OLYMPUS Biological Confocal Laser Scanning Microscope FV1200.

### Fluorescence *In Situ* Hybridization (FISH)

Xenomas were fixed in 4% paraformaldehyde, decolorized with 6% H_2_O_2_ in ethanol for 2 h, washed for four times (10 min each) with 0.01M PBS buffer (pH 7.2). The samples were then incubated with DAPI at 37°C for 30 min to stain the nuclei. After washing four times (5 min each) in 0.01 M PBS buffer (pH 7.2), the slides were suspended with Fluoromount™ Aqueous Mounting Medium (Sigma) and mounted with a cover glass. Based on the 16S rRNA sequence of *V. necatrix* BM, a DNA probe, VnLSU-V1-Cy3 (5’-Cy3-GTATTCTATTACGACCTTC-3’), was designed using Primer3 software (http://fokker.wi.mit.edu/primer3/) and checked the specificity using the Ribosomal Database Project II “probe match” analysis tool (Gottlieb et al., 2006). Besides, the probe specificity was experimentally verified in silkworm BmE cells infected by *V. necatrix* and *N. bombycis*, the latter of which was labeled with a multiclonal antibody as described in (Song et al., 2020). Stained samples were wholly mounted and viewed under an OLYMPUS Biological Confocal Laser Scanning Microscope FV1200.

### Transmission Electron Microscope (TEM)

TEM was performed as previously described (Wu et al., 2010) with slight modifications. Xenomas were fixed with 2.5% glutaraldehyde for 2 h, and washed four times (15 min each) with 0.1 M PBS buffer (pH 7.2), then fixed for 2 h with 1% osmium tetroxide and washed for four times (15 min each) with 0.1 M PBS buffer (pH 7.2). Subsequently, the samples were dehydrated two times with gradient ethanol and 100% acetone, infiltrated with gradient Epon812 (SPI, USA) resin, buried with 100% resin, and aggregated for 48 h at 70°C. Ultrathin sections were made using a LEICA EM UC7 ultra microtome. The sections were stained with 3% uranyl acetate for 20 min, followed by lead citrate for 15 min. The dyed sections were rinsed for six times with distilled H_2_O, naturally dried, and then photographed with a JEM-1400 Plus TEM under 80 kv acceleration voltage.

## Results

### The Development of the Xenoma

**Figure 1:**
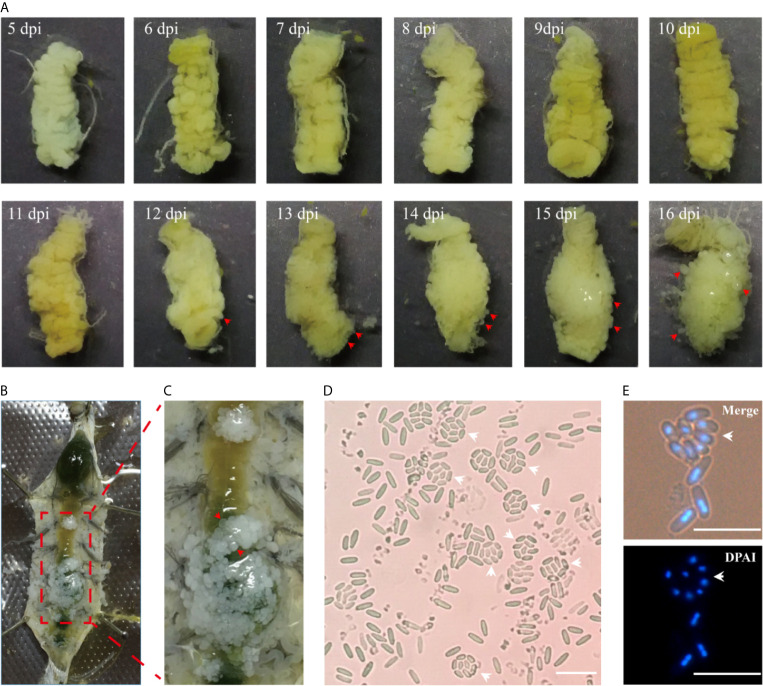
The development of xenoma on the midgut of silkworm infected by *V. necatrix* BM. **(A)** The silkworm midgut from 5 to 16 dpi. The xenomas (arrowhead) could be observed after 12 dpi and were obvious after 13 dpi. The midgut was surround by massive xenomas after 16 dpi; **(B)** The infected silkworm larva was dissected in the 5^th^ instar; **(C)** Massive whitish xenomas (arrowhead) were shown on the outer surface of the infected midgut; **(D, E)** The *V. necatrix* BM in a xenoma produced a large number of meiospores (octospores) contained within a sporophorous vesicle (arrowhead). The bar indicates 10 µm.

The midgut is the main digestive organ of silkworm, and also the first organ infected by microsporidia. Silkworms orally inoculated in the third instar showed no obvious xenoma in 11 days post infection (dpi) (**Figure 1A**). On the 12^th^ dpi, a few of xenomas were observed on the posterior of midgut. On the 13^th^ dpi, the midgut showed a heavier infection and became whitish on the posterior, suggesting that a large number of little xenomas were forming. Subsequently, the xenomas grew larger, and the intestinal enlargement became evident on the 16^th^ dpi. The anatomy showed that a large number of xenomas formed on the posterior of the midgut (**Figures 1B, C**). This particular parasitic pattern is known as an xenoma in many aquatic animals infected by microsporidia. The *V. necatrix* xenoma is spherical in shape and 1 to 5 mm in size (Luo et al., 2014; Meng et al., 2018). Microscopic observations of the xenoma manifested a great many pathogens in different stages and some vesicles each containing eight monocytic spores, the octospores (**Figures 1D, E**).

### The Development of the Pathogens in Xenoma

**Figure 2:**
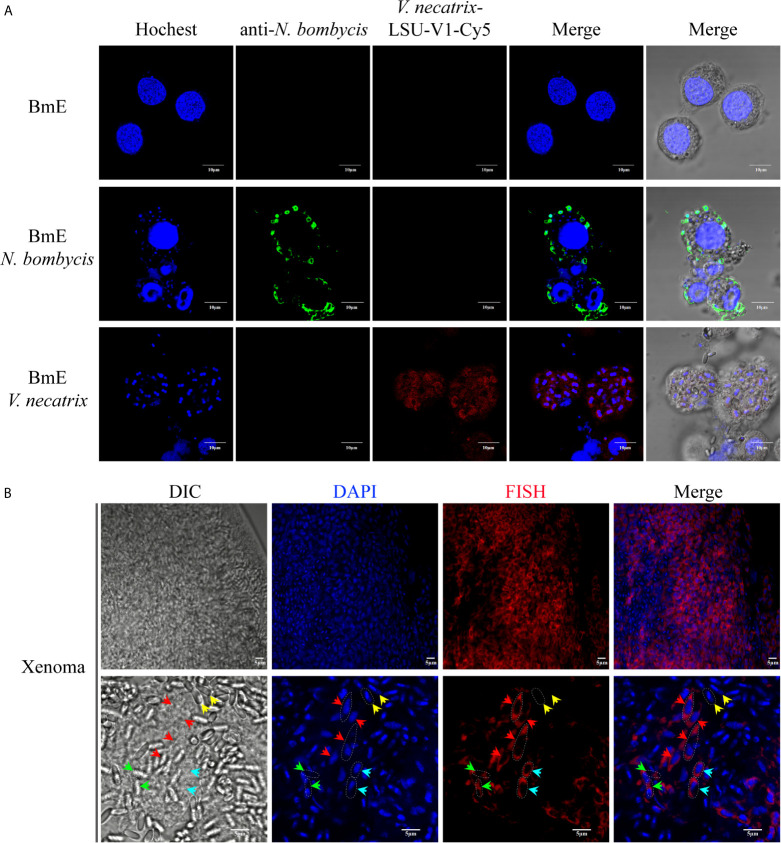
FISH and DAPI staining of *V. necatrix* BM in xenoma. **(A)** The parasite purity was verified by FISH with a probe of *V. necatrix* ribosomal RNA (red) and IFA using an antibody against *N. bombycis* (green) in infected BmE cells, respectively. Bar, 10 µm. **(B)** The nucleus of *V. necatrix* BM in all stages was stained with DAPI (blue). The parasites in development were labeled using FISH with a probe of the ribosomal RNA (red). Red arrowhead, meront; Cyan arrowhead, sporont; Yellow arrowhead, mature spore; Green arrowhead, empty (germinated) spore; Bar, 5 µm.

The *V. necatrix* BM in the xenoma was demonstrated by FISH for labeling pathogens in proliferation and DAPI for staining all nuclei. The specificity of the FISH probe and purity of the parasites were firstly verified in silkworm BmE cells infected by *N. bombycis* and *V. necatrix*. In result, the *V. necatrix* was specifically labeled by the probe, while there was no probe sign found in *N. bombycis*, which was instead demonstrated by the specific antibody (**Figure 2A**). As shown in **Figure 2B**, the xenoma was full of parasites in proliferative and mature stages. The meronts were transparent and not visible under DIC, but specifically labeled by FISH and DAPI, which stained the cytosol in red and the nucleus in blue, respectively. The meronts were fusiform in shape and much longer than any other stages for reaching 10 μm in length. The meront nucleus was also much larger, and showed lighter DAPI fluorescence compared with that of the mature spores, indicating that meront chromatins were likely in highly active state. The sporont, clearly stained by FISH and DAPI, were shown oval in shape and 5 μm in length, and became recognizable under DIC for the outer wall being slightly light-reflecting. Large quantities of mature spores were observed in xenoma, especially in the central area. The mature spores could not be labeled by FISH probes for being coated with thick spore wall but showed strong DAPI signals, which manifested condensed nuclei. Mature spores displayed high refractivity and clear outline under DIC so that they were easily recognized under light microscopy. Moreover, some germinated and empty spores were also observed under DIC, which showed no FISH and DAPI signals, suggesting that autoinfection happened inside the xenoma. Besides, the large numbers of germinated spores also indicated that the parasites were in massive reproduction.

### The Morphology of the Xenoma

**Figure 3:**
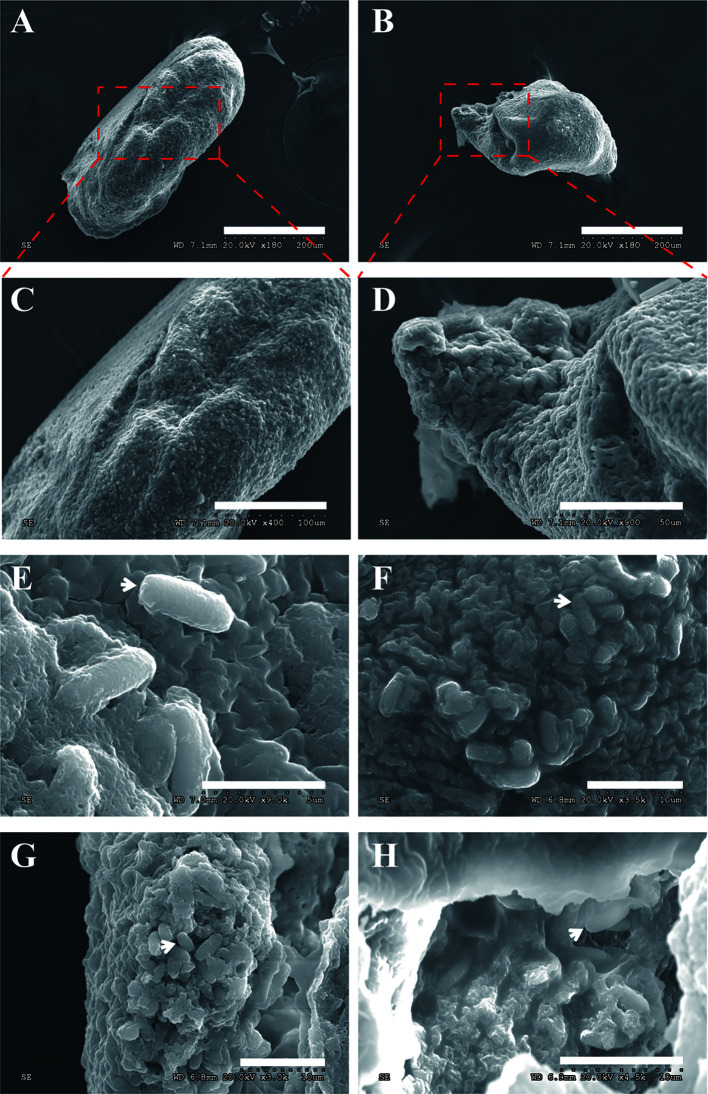
The xenoma observed by SEM. **(A, B)** The elliptical and spindle intact xenoma. **(C, D)** The enlarged graph of the dotted box in panels **(A, B, E)** The mature spores (arrowhead) adhering to the surface of the xenoma, the bar is 50 µm. **(F)** The mature spores inlaid on the surface of the xenoma, the bar is 10 µm. **(G, H)** The mature spores embedded in a transverse xenoma. Bar, 10 µm.

Manifested by the SEM, the xenoma was long oval and spindle in shape (**Figures 3A, B**). The outer surface of the xenoma was covered by highly dense protrusions (**Figures 3C, D**), which were shown to be mature spores by the enlarged views (**Figures 3E, F**). Some spores looked like floating on and adhering to the xenoma surface, and some were partially inlaid in xenoma wall, suggesting that the spores were exiting from the xenoma, and the xenoma could be a source for infecting other tissues. The internal structure was also observed from the broken xenoma using SEM and showed a lot of mature spores embedded in the loose matrices inside (**Figures 3G, H**).

### The Organelles in the Xenoma

**Figure 4:**
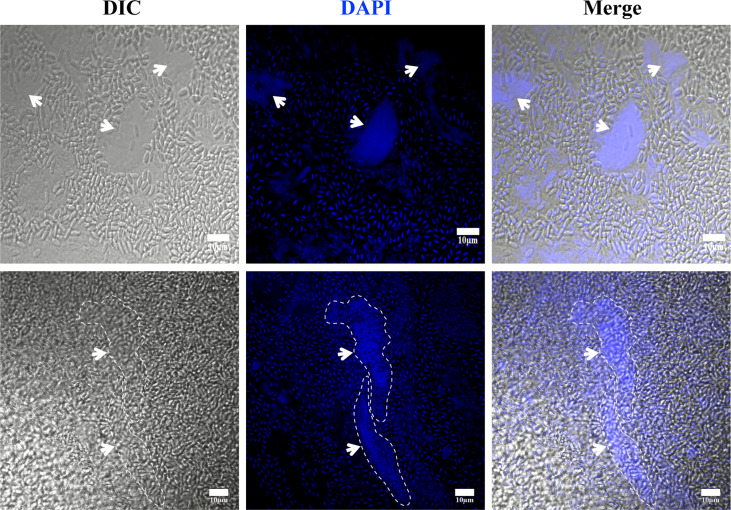
The observation of xenoma nucleus. The nucleus (arrowhead) of xenoma and *V. necatrix* BM were stained with DAPI (blue). The hypertrophied nucleus was labeled with a dashed line. Bar, 10 µm.

The *V. necatrix* xenoma is a membrane-encapsulated cystic structure that forms in muscle tissue. However, it is unclear whether there is a host nucleus in the xenoma. In the DAPI stained xenoma, besides a great number of pathogens nuclei, multiple host nuclei were observed (**Figure 4**). These host nuclei were surrounded by massive pathogens and much larger than common nucleus, and apparently hypertrophic deformed and branched and lobed (dash line in **Figure 4**). The multinuclear feature suggested that the xenoma likely produced by fusion of multiple muscle cells.

Microsporidian genomes are compact and reduced, and have lost most genes responsible for the *de novo* synthesis of nucleotides, amino acids, and lipids (Corradi et al., 2010; Heinz et al., 2012; Nakjang et al., 2013). Instead, microsporidia evolved strategies to regulate host pathways for obtaining nutrients from host (Bernal et al., 2016; Han et al., 2020). Massively proliferating in xenoma, the parasites would get large quantities of nutrients from host. Therefore, it is interesting to see what happens to the xenoma mitochondria and endoplasmic reticulum (ER), which are vital organelles in the synthesis of nutrients and energy.

**Figure 5:**
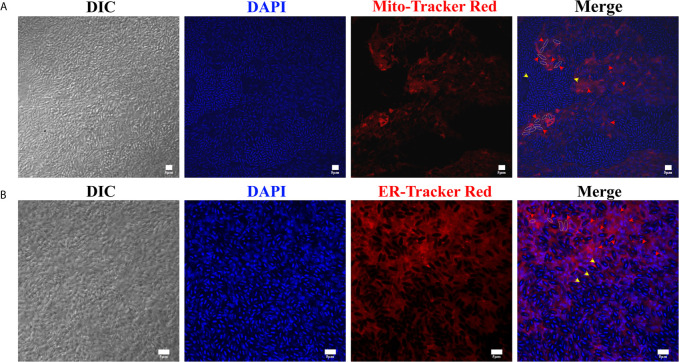
The observation of xenoma mitochondria and endoplasmic reticulum (ER). **(A)** The xenoma was stained with Mito-Tracker Red for labeling mitochondria (red) and DAPI for dying nucleus (blue). **(B)** The xenoma was stained with ER-Tracker Red for labeling ER (red) and DAPI. The nucleus of meronts (red arrowhead) and spores (yellow arrowhead) were stained with DAPI (blue). Bar, 5 µm.

Herein, the mitochondria and ER in the xenoma were stained using Mito-Tracker and ER-Tracker, respectively. In result, the mitochondria were shown in red and mainly appeared and highly aggregated in areas full of meronts but showing less mature spores (**Figure 5A**). The densely aggregated mitochondria showed no distinct outline, instead looked like linking up into a single stretch, suggesting that the replication of mitochondria was significantly increased. Massive mature spores, each with two nuclei, were conspicuous under DIC and DAPI staining, aggregated and formed clusters. The ER was also marked in red by ER-Tracker and densely distributed in the xenoma, and even denser in the locations where pathogens were in proliferation (**Figure 5B**). The high density of mitochondria and ER around the proliferative pathogens suggested that the xenoma likely supplied abundant energy and nutrients for the proliferation of the parasites.

### The Subcellular Structures of the Xenoma

**Figure 6:**
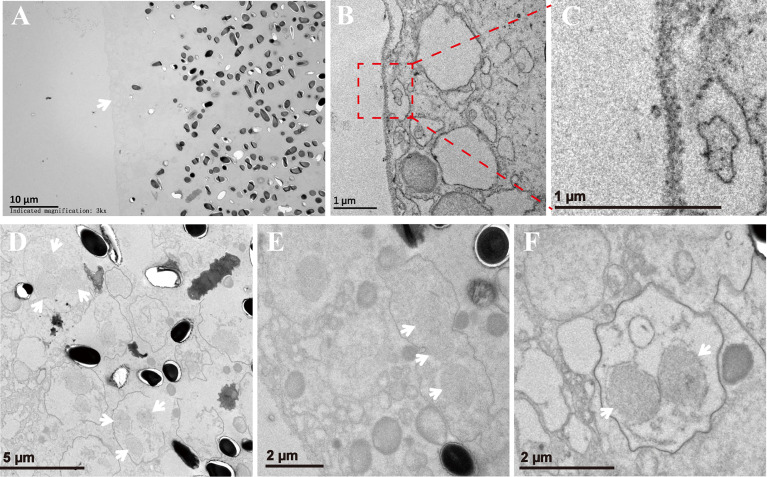
The xenoma observed by TEM. **(A)** The outer wall of xenoma (arrowhead) and *V. necatrix* BM inside. **(B, C)** The magnified outer wall of the xenoma. **(D–F)** The development of *V. necatrix* BM in xenoma. The arrowhead indicates the nuclei of the parasites.

The xenoma was analyzed using TEM to observe the ultrastructure of the organelles and pathogens inside. The outer wall of the xenoma was a thin and single-layer membrane for about 100 nm (**Figures 6A–C**). The xenoma interior was full of vesicle structures and pathogens. Pathogens in the early xenoma were nearly in proliferative stages, most of which were merogony (**Figures 6D–F**). The meronts usually contained two to three nuclei, and surrounded by many vesicles in high or low electron density. The mature spores with a thick wall manifested high electron density.

## Discussion

*V. necatrix* can infect a wide range of lepidoptera (Maddox et al., 1981). Besides in silkworm, the proliferative morphology of *V. necatrix* in others lepidopteran insects was also characterized (Maddox et al., 1981; Moore and Brooks, 1992; Luo et al., 2014). A dominant feature of the *V. necatrix* infection is that it can rapidly multiply and quickly kill the host. The maximum spore production of 1 × 10^10^ spores/g of host was obtained in *Helliothis zea* and *Trichopluisia ni* (Maddox et al., 1981). The spore production and lethal period depend on multiple factors, including the pathogen genotype, host species, temperature, inoculation dosage, and larval instar challenged. When infecting silkworm with a dosage of 1 × 10^4^ V*. necatrix* spores per larva in fourth instar, we obtained an average of 4.52 × 10^8^ spores from a fifth-day pupa (**Supplementary Table 1**, **Supplementary Figure 1**).

The xenoma is a common pathological structure made by some microsporidian species and frequently reported in aquatic animals (Lom and Nilsen, 2003; Lom and Dykova, 2005). The tissue that xenoma produced from is varied in different hosts. In silkworm infected by *V. necatrix*, the xenoma is made from muscle cells and grows on the outer surface of midgut. In *Lophius piscatorius* infected by *Sprague* sp., the xenoma is formed in nerve tissue (Campbell et al., 2013). In *Endoreticulatus eriocheir*-infected crab, the xenoma was found in hepatopancreas (Ding et al., 2016). The xenoma made by *A. portucalensis* was also found in hepatopancreas of the common foreshore crab and contained a great many of cysts consisting of hypertrophic host cells (Azevedo, 1987). The varied locations of the xenoma reflect the tissue preference of different microsporidia. Besides, in the late stage of development, a plurality of small xenomas were formed in a developed (or mature) xenoma, and there were some pinocytotic vesicles in the center of xenoma, which are probably the secondary xenomas formed inside the primary ones (Lom and Dykova, 2005).

The *V. necatrix* in the xenoma presented a binary life cycle, the *Nosema*-like (type species: *N. bombycis*, Nägeli, 1857) and *Thelohania*-like (type species: *T. giardia*, Henneguy and Thelohan, 1892). The *Nosema* presents two nuclei in all stages of the life cycle (Vávra, 1976), while the *Thelohania* develops monokaryotic within a sporophorous vesicle to form octospores (Jouvenaz, 1984). It was reported that the octosporoblastic sporogony occurred primarily at low temperatures (Moore and Brooks, 1992), indicating that the life cycle of some microsporidia can be regulated by temperature.

Wrapped by a host membrane, the intact xenoma provides an environment free of host immune surveillance for that the pathogen antigens cannot be exposed at the surface (Dykova et al., 1980; Canning and Curry, 2005). Moreover, the xenoma contributes to parasite proliferation by generating massive nutrients and energy. The *V. necatrix* xenoma on the silkworm midgut is generated from muscle cells, which were specialized and transformed into a powerful cyst containing multiple hypertrophic nuclei and fully filled with ER and mitochondria around the proliferative parasites. These modified organelles could provide the parasites with much more energy and nutrients (Canning and Curry, 2005). The xenoma nucleus is hypertrophic and branched or lobed. This pathological feature is similar to that of the xenoma produced by fish microsporidia (Lom and Dykova, 2005; Azevedo et al., 2016), however its function and mechanism remain illumination.

*V. necatrix* and *N. bombycis* are phylogenetically close to each other and natural pathogens infecting silkworm (Liu et al., 2012; Luo et al., 2014). However, both pathogens are quite different in spore morphology and pathology. *V. necatrix* can high-efficiently produce dikaryotic large spores and unicellular small octospores in the xenoma, while *N. bombycis* does not make xenoma and only generates dikaryotic and uniform size spores. On the other hand, *N. bombycis* infects all silkworm tissues, and horizontally and vertically transmit by invading ovary and oocyte. Nevertheless, *V. necatrix* is able to infect nearly all silkworm tissues except for the ovary so that cannot be vertically transmitted (Meng et al., 2018). These variances are important factors that lead to different virulence and transmissive efficiency between the two parasites.

In summary, the xenoma produced by *V. necatrix* BM is a specialized syncytium with increased mitochondria and ER and hypertrophic nuclei to promote the production of energy and nutrients for the massive the massive proliferation of the parasites inside. Our work provides a clearer view of the xenoma made by *V. necatrix* in silkworm.

## Data Availability Statement

The original contributions presented in the study are included in the article/**Supplementary Material**. Further inquiries can be directed to the corresponding authors.

## Author Contributions

TL and ZZ contributed to conception and design of the study. ZF, QH, CW, XM, and BY contributed to experimental analysis. TL and ZF contributed to data analysis. TL and ZF wrote the first draft of the manuscript. All authors contributed to the article and approved the submitted version.

## Funding

This work was supported by grants from the National Natural Science Foundation of China (31772678, 31770159, and 31472151), Natural Science Foundation of Chongqing, China (cstc2019yszx- jcyjX0010).

## Conflict of Interest

The authors declare that the research was conducted in the absence of any commercial or financial relationships that could be construed as a potential conflict of interest.

The reviewer JX declared a shared affiliation, with no collaboration, with one of the authors, ZZ, to the handling editor at the time of review.

## Supplementary Material

Supplementary Figure 1 Counting spores with a blood counting chamber. *V. necatrix* BM spores were purified from the fifth-day pupae for counting the spore production. The spores were diluted ×500 and added to the counting chamber. Spores in five middle-sized grids in the four corners and center were counted.
