# Supplementary figures and images for: Characterizing the Xenoma of Vairimorpha necatrix Provides Insights Into the Most Efficient Mode of Microsporidian Proliferation
Source: Front Cell Infect Microbiol. 2021 Jun 16;11:699239. doi: 10.3389/fcimb.2021.699239 (PMC8242933; doi:10.3389/fcimb.2021.699239)

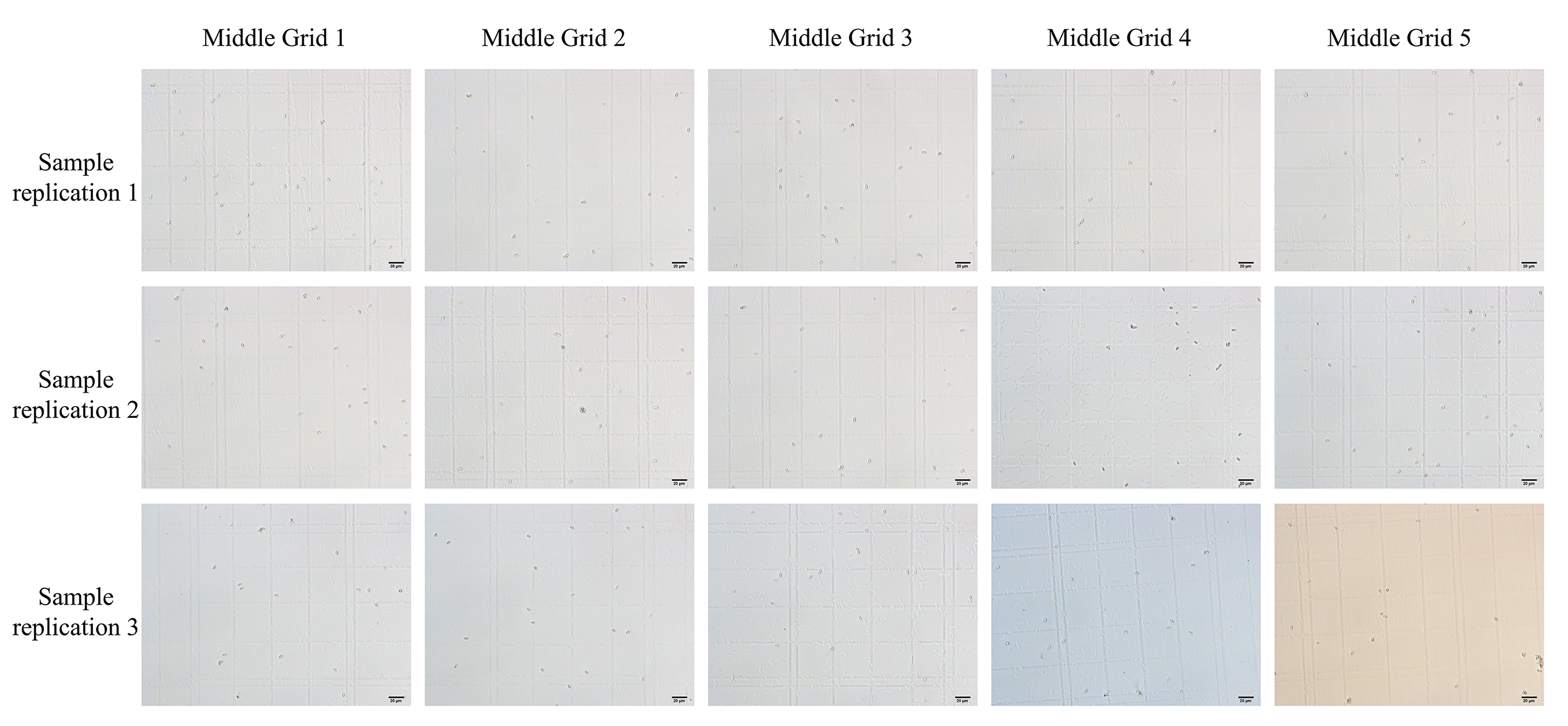

Supplement: Supplementary Figure 1 — Counting spores with a blood counting chamber. V. necatrix BM spores were purified from the fifth-day pupae for counting the spore production. The spores were diluted ×500 and added to the counting chamber. Spores in five middle-sized grids in the four corners and center were counted. [file Image_1.tif]
